# The Thirty-Fifth Annual Commencement of the Baltimore College of Dental Surgery

**Published:** 1875-03

**Authors:** 


### EDITORIAL. ETC.

---

*The Annual Commencement of the Baltimore College of Dental Surgery.*—The *Thirty-fifth* Annual Commencement of this institution was held at the Concordia Opera House, on Thursday evening, February 25th, and despite the inclement weather the extensive auditorium was filled with a brilliant audience, composed largely of ladies. The Stage, as usual on such occasions, was occupied by the Faculty of the College, members of the Graduating Class, and invited guests, among the latter being prominent clergymen, professors of the different medical schools of the city, and medical and dental practitioners of Baltimore and distant places.

The exercises commenced with a march by the Fifth Regiment Orchestra, A. Itzel, Leader, during which the Faculty and members of the Graduating Class entered and occupied their seats upon the Stage, when Rev. Dr. William T. Brantly, of the Seventh Baptist Church, delivered a very impressive prayer.

The Dean of the Faculty, Professor F. J. S. Gorgas, then read the act of the Legislature of the State of Maryland, authorizing the Baltimore College to confer the degree of Doctor of Dental Surgery, and announced the names of the graduates, seventeen in number.

After music by the Orchestra, which finely rendered a number of operatic and popular selections during the evening, the Degree of "Doctor of Dental Surgery" was conferred by Professor Gorgas upon the following gentlemen, who had been subjected to very rigid examinations on Dental Surgery, Therapeutics and Materia Medica, Physiology and Pathology, Chemistry, Dental Mechanism, Anatomy, and Clinical Dentistry:

SUBJECT OF THESIS.

Reverdy Brook Beall, *Maryland.*—Mechanical Dentistry.
Charles Campbell, *Maryland.*—Decay of the Teeth.
John Ernest McBean Chevers, *West Indies.*—Dental Mechanism.
Joel Beverly Coyle, *Georgia.*—Diseases of the Teeth.
Erastus Clarence Eversole, *Virginia.*—Artificial Teeth.
Charles Denny Hilliard Fort, *Mississippi.*—Dentition.
James Orlando Hodgkin, *Virginia.*—Dental Hygiene.
Hardy Miles Hunter, *Texas.*—Dentarius.
James Murphy King, *Tennessee.*—The Teeth.
Charles Luther Moore, *Georgia.*—Dental Prosthesis.
Charles James Phillips, *California.*—Artificial Dentures.
Samuel Dillard Rambo, *Georgia.*—Anæsthetics.
George Bangheart Raub, *New Jersey.*—Dental Caries.
Robert Edward Sparks, *Canada.*—Nitrous Oxide Gas in Dental Operations.
I. Hamilton Thomas, *Virginia.*—Filling Teeth.
Edward Franklin Wayman, *Texas.*—The Circulation.
Garner Brown White, *South Carolina.*—Dental Caries.

After receiving their diplomas the graduates were the reci ients of a large number of handsome boquets and vases ot natural and wax flowers. The Dean, Prof. F. J. S. Gorgas, was presented with a magnificent cross of wax flowers, enclosed in a large glass case, and which was universally admired.

The Valedictory Address was then delivered by Professor Thomas S. Latimer, M. D., in which he endeavored to impress upon the graduates the value of labor, true manhood, high aims and generous purposes, and above all, loving hearts, pure lives, and readiness at all times to abandon error and follow the guidance of truth, forcibly expressing the noblest sentiments and incentives that should govern professional life. He also referred to the fact that happiness did not consist in making money alone, and advised them to act well their parts and not count too much on human praise It was an able and edifying address, and at its close, the speaker was presented with several beautiful boquets of flowers.

The Valedictory was followed by the Class Address, delivered by James Murphy King, of Bristol, Tennessee, a member of the Graduating Class, which for easy and effective delivery and elegance of composition, has never been excelled on any former occasion. Dr. King commenced with expressing his thanks for the large attendance, then referred to the advantages offered by the Baltimore College of Dental Surgery to students, that it was the oldest institution of the kind in the world, and remarked that through its aid the science of Surgery had been rendered available to women. The speaker also expressed warm thanks to the Faculty for the sympathy and aid always generously extended to the students, and sketching the part of professional duty, pointed out the hill-tops in the march of life toward the temple of distinction, closing with an affectionate farewell to his fellow graduates and class mates. Both the Valedictory and Class Address will appear in the April number of this Journal.

After a medly overture, including Home Sweet Home and Dixie, by the Orchestra, the exercises closed with a benediction by the Rev. Dr R. Hammond

The President of the Graduating Class was Charles L. Moore, of Griffin, Georgia; Secretary, Jas. M. King, of Bristol, Tenn.;

Treasurer, J. O. Hodgkin, of Warrenton, Va; and the Committee of Arrangements, S. D. Rambo, C. D. H. Fort, H. M. Hunter, Charles Campbell, J. B. Coyle, E. F. Wayman, G. B White, J. E. Chevers and R. E. Sparks.

Every specimen set of teeth presented by the candidates for graduation, was constructed of metal' and although manipulation in all the different forms of plastic work is taught in the Baltimore College, not a single rubber set was presented with the theses.

The concluding lectures of the past session and Infirmary demonstrations, were held in the new College building, S. E. Cor. Eutaw and Lexington Sts., which for elegance and convenience, far surpasses any institution of the kind in existence.

The Thirty-sixth Annual Session, will commence on the 14th of October, 1875, and continue until March, in 1876.

The Infirmary is open during the entire year for dental operations.

---

